# Outcomes of Radical Radiotherapy in Prostate Cancer Patients Over 80 Years of Age

**DOI:** 10.7759/cureus.93811

**Published:** 2025-10-04

**Authors:** Andrew Mbencho, Syed Owais Bokhari, Kyaw Z OO, Joachim Chan, Helen Wong, Zaf Malik, Richard Walshaw, Isabel Syndikus

**Affiliations:** 1 Clinical Oncology, The Clatterbridge Cancer Centre NHS Foundation Trust, Liverpool, GBR; 2 Medical Oncology, The Clatterbridge Cancer Centre NHS Foundation Trust, Liverpool, GBR; 3 Digital Services, The Clatterbridge Cancer Centre NHS Foundation Trust, Liverpool, GBR

**Keywords:** older patient, prostate cancer, radical treatment, radiotherapy (rt), recurrence

## Abstract

Background

Despite advances in surgery, radiotherapy, and systemic therapies, treatment selection and sequencing remain major challenges in optimizing outcomes for patients with prostate cancer. Men aged 50-69 years with screen-detected disease often achieve favorable outcomes across various treatment strategies, whether managed with active monitoring, prostatectomy, or radical radiotherapy. However, limited information is available on outcomes in older patients. This study retrospectively evaluates survival outcomes in older patients treated with radical radiotherapy at a tertiary UK cancer center.

Methods

At Clatterbridge Cancer Centre NHS Foundation Trust, the electronic health records system was used to identify patients aged 80 years or older who received radical radiotherapy over a five-year period, with at least five years of follow-up. Demographic data collected included disease risk profile and comorbidities. Biochemical recurrence-free survival (BFS), metastasis-free survival (MFS), hormone-free survival (HFS), and overall survival (OS) were analyzed.

Results

Between January 2013 and December 2017, 82 patients were treated and analyzed. The median age was 81 years, and most patients had a performance status of 0 or 1 with minimal significant comorbidities. Prostate cancer was classified as high or very high risk (National Comprehensive Cancer Network) in 88% of patients. Five-year BFS, MFS, HFS, and OS were 77%, 88%, 86%, and 77%, respectively. Despite receiving radical radiotherapy, 21% of deaths were attributable to prostate cancer.

Conclusions

Prostate cancer patients aged 80 years or older treated with radical radiotherapy can achieve favorable BFS and HFS despite high-risk disease. These outcomes are clinically meaningful, as untreated disease may lead to worsening symptoms and the future need for lifelong hormone therapy with its associated toxicities. Nonetheless, a substantial proportion of patients will die from the disease despite receiving radical radiotherapy.

## Introduction

Prostate cancer predominantly affects older men, with incidence increasing with age. Rates rise rapidly from around 45 years of age, peaking within the 75-79 age group, after which they decline slightly and remain generally stable in the oldest age groups [1]. This plateau is likely due to reduced diagnostic activity in the very elderly, including fewer prostate-specific antigen (PSA) tests, less frequent biopsy referrals, and declining incidental detection from TURP (transurethral resection of the prostate), as medical management of benign prostatic hypertrophy has become more common [2].

In the previous year, the 15-year outcomes from the ProtecT study, which compared outcomes in younger men (aged 50-69 years) in the UK with localized prostate cancer randomized to active monitoring, prostatectomy, or radical radiotherapy, were published, showing that prostate cancer-specific mortality was low regardless of the treatment assigned [3]. Clinical trial participants tend to be younger and less frail than those routinely encountered in clinical practice, who often have higher risks from competing comorbidities that may reduce the absolute benefits of prostate cancer treatment [4-9]. Given that life expectancy for men in the UK is 78 years, it is important to consider the merits of active treatment in older patients. Therefore, a retrospective study was performed at our tertiary UK cancer center to evaluate outcomes in patients aged 80 years or older treated with radical prostate radiotherapy.

## Materials and methods

This retrospective study was conducted at Clatterbridge Cancer Centre NHS Foundation Trust and approved by the Institutional Research and Audit Committee. The institutional electronic health records system (Meditech, Medical Information Technology, Inc., Westwood, Massachusetts, USA) was used to identify all male patients aged 80 years or older who underwent radical prostate radiotherapy for localized prostate cancer over a five-year period. To be included in the analysis, patients were required to have at least five years of clinical follow-up data available from the date of treatment initiation.

Data were extracted regarding patient demographics, tumor characteristics, and comorbidities. Information was collected on risk profile (TNM staging, PSA, and Gleason score) and the National Comprehensive Cancer Network (NCCN) risk stratification [10]. The Charlson Comorbidity Index (CCI) [11] was retrospectively calculated for each patient based on documented comorbid conditions at the time of radiotherapy to assess baseline health status and competing risk.

Biochemical recurrence-free survival (BFS) and metastasis-free survival (MFS) were calculated from the date of the first radiotherapy fraction to the date of PSA rise from nadir by ≥ 2.0 ng/ml (Phoenix definition of biochemical failure) and to the date of radiological identification of metastases, respectively [12].

For patients who relapsed, the subsequent treatment of choice was recorded. Hormone-free survival (HFS) was calculated from the date of the first radiotherapy fraction to the date that palliative hormone therapy was initiated for recurrent disease. For patients who had died, overall survival (OS) was calculated from the date of the first radiotherapy fraction to the date of death. Cause of death was obtained from death certificates or, if unavailable, adjudicated by the study investigators using medical records and available investigation results. For patients alive at the time of data analysis, follow-up duration was measured from the date of the first radiotherapy fraction to the most recent oncology clinic appointment or the latest recorded PSA result, whichever was later.

All data were anonymized prior to analysis to ensure patient confidentiality.

## Results

Between January 2013 and December 2017 inclusive, 84 patients aged 80 years or older received radical prostate radiotherapy (Table 1). Two patients were omitted from the analysis because they were enrolled in clinical trials and received non-standard-of-care treatment at the time: one in the PACE B trial, who received prostate stereotactic ablative radiotherapy (SABR), and one in the STAMPEDE trial, who received adjuvant androgen receptor-targeted agents.

**Table 1:** Baseline demographics

Characteristic	Median (range)
Number of patients	82 patients
Age	81 years (80-87 years)

Of the 82 eligible patients, the median age was 81 years (range, 80-87). Most had a performance status of 0 or 1 (85% of patients; Table 2), and the majority had a CCI score of 7 or less (91% of patients; Table 3).

**Table 2:** Performance status [13]

Performance status	Number of patients
0	40
1	30
2	4
Unspecified	8

**Table 3:** Comorbidities The CCI score includes age and cancer diagnosis. CCI, Charlson Comorbidity Index [11]

CCI	Number of patients
6	50
7	25
8	6
Unavailable	1

For one patient, initial comorbidity data were inaccessible. The majority of patients had high- or very high-risk prostate cancer according to NCCN risk stratification (88% of patients; Table 4).

**Table 4:** NCCN risk stratification NCCN, National Comprehensive Cancer Network [10]

NCCN risk stratification	Number of patients
Intermediate	10
High or very high	72

Seventy-one patients received prostate-only radiotherapy, of whom 43 received 72 Gy in 32 fractions, and, following publication of the CHHiP trial, 28 received 60 Gy in 20 fractions (Table 5). The prostate and pelvic nodal radiotherapy group (11 patients, two of whom had pelvic nodal involvement on staging) received 72 Gy in 32 fractions to the prostate, 64 Gy to involved nodes, and 50 Gy to uninvolved nodes (Table 6). All patients also received concurrent hormone therapy for between six months and three years, according to clinician preference.

**Table 5:** Radiotherapy to prostate

Radiotherapy fractions	Number of patients
72 Gy in 32 fractions	43
60 Gy in 20 fractions	28

**Table 6:** Radiotherapy to prostate and pelvic nodes

Radiotherapy fractions	Number of patients
72 Gy in 32 fractions	11
60 Gy in 20 fractions	0

The median duration of follow-up was 72 months (range, 2-120 months).

**Figure 1:**
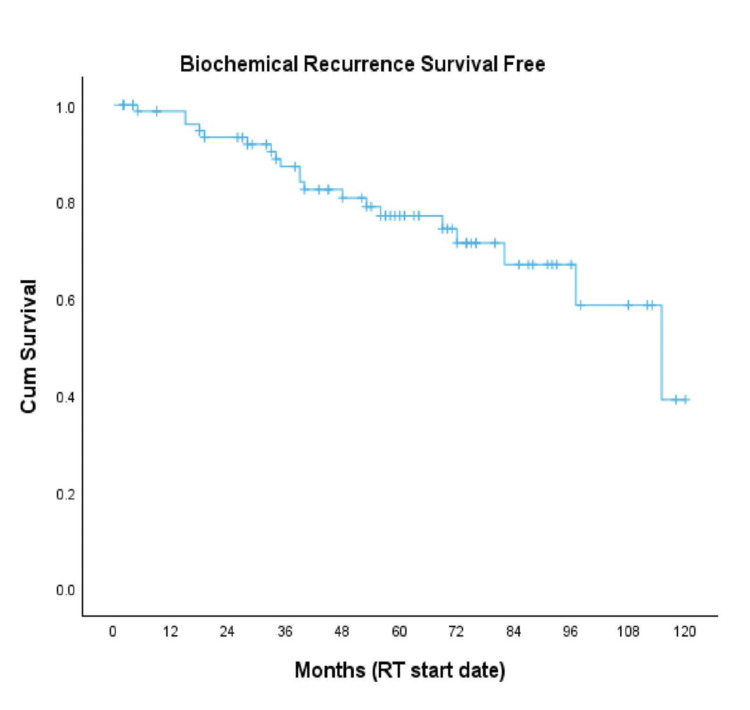
Kaplan-Meier curve for BFS BFS, biochemical recurrence-free survival; RT, radiotherapy [14]

Twenty patients (24%) experienced biochemical recurrence. The five-year BFS rate was 77%, and the median BFS was 115 months (Figure 1).

**Figure 2:**
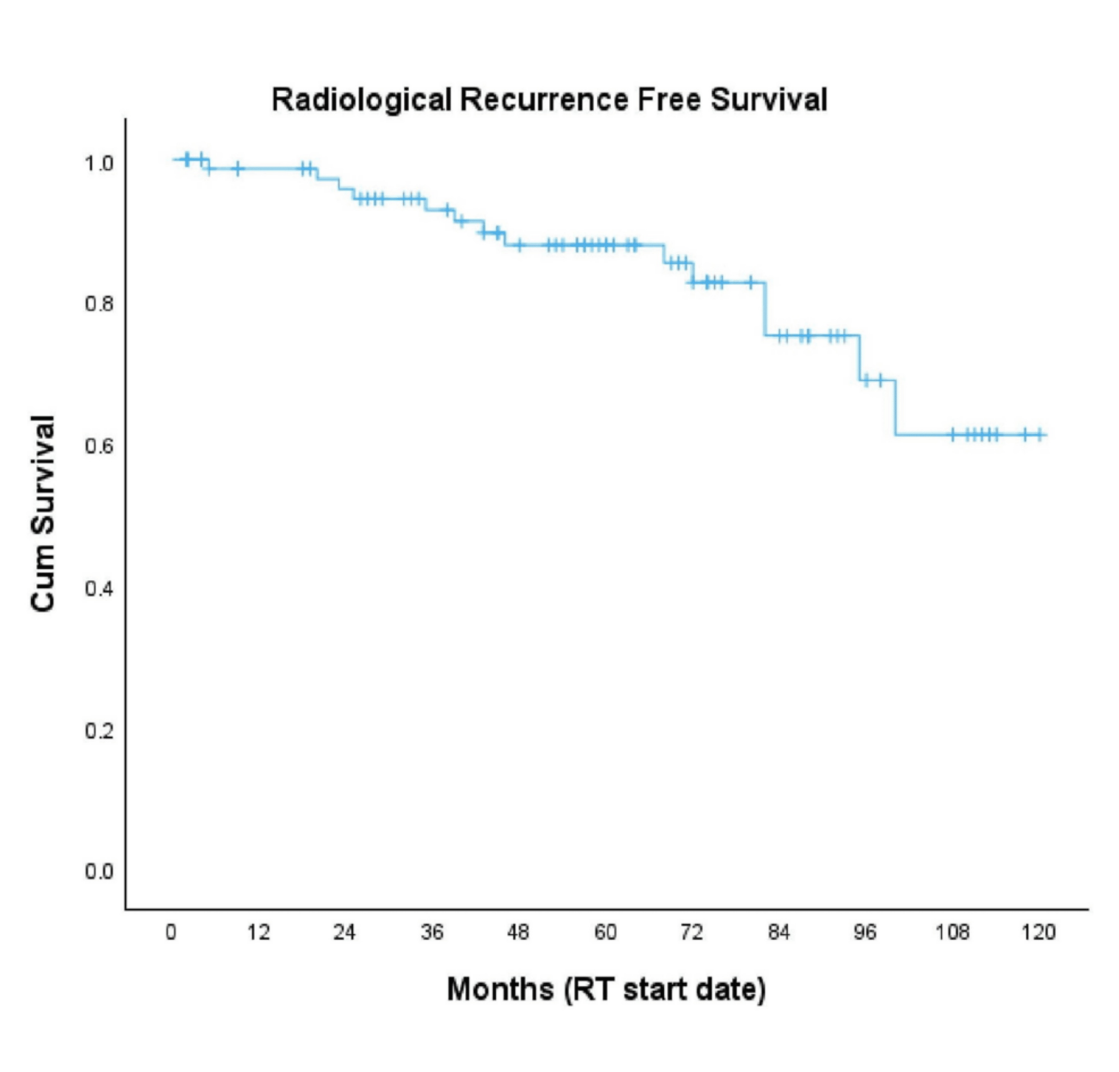
Kaplan-Meier curve for MFS MFS, metastasis-free survival; RT, radiotherapy [14]

Fourteen patients (17%) developed metastatic recurrence. The five-year MFS rate was 88%, and the median MFS was not reached (Figure 2).

**Figure 3:**
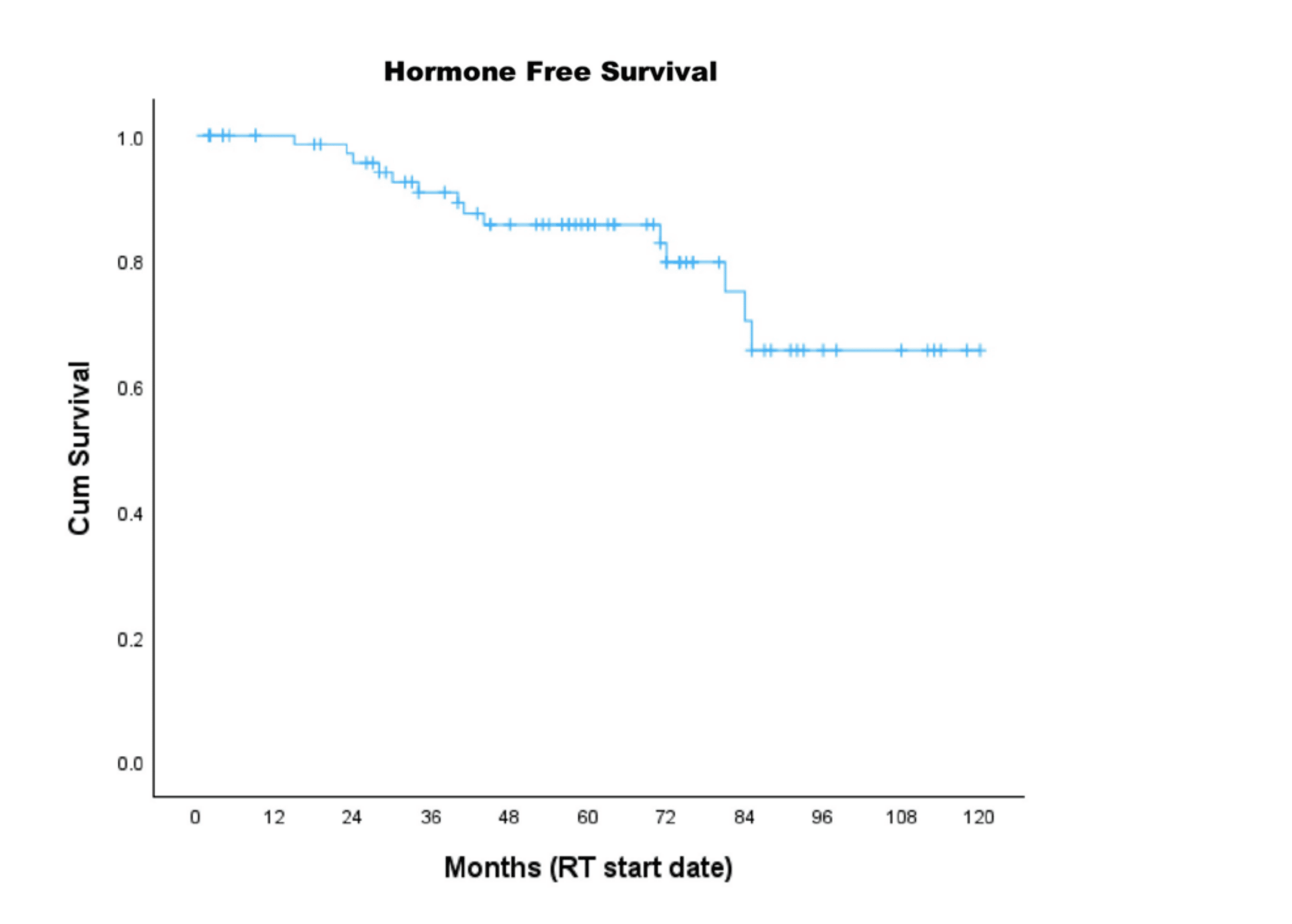
Kaplan-Meier curve for HFS HFS, hormone-free survival; RT, radiotherapy [14]

Overall, 17 of 82 patients received subsequent therapy, including one who underwent SABR to a pelvic lymph node. Sixteen patients (20% of all patients) initiated palliative hormone therapy: one developed bone metastasis during adjuvant hormone therapy, six received therapy for biochemical recurrence only, and nine received therapy for both biochemical and radiological recurrence. The five-year HFS rate was 86%, and the median HFS was not reached (Figure 3).

In the palliative first-line setting, most patients received luteinizing hormone-releasing hormone (LHRH) therapy alone (11 of 16 patients), while three received bicalutamide alone, one received maximal androgen blockade, and one received LHRH with abiraterone acetate in the metastatic hormone-sensitive setting (16 cycles). In the hormone-relapsed setting, six patients received advanced hormone therapy (five received abiraterone acetate, median 6 cycles (range, 1-29); one received enzalutamide, 11 cycles). Of the seven patients who received advanced hormone therapy, five died, with a median OS of 75 months (range, 48-112 months) from the date of the first radiotherapy fraction; four died from prostate cancer and one from heart failure.

**Figure 4:**
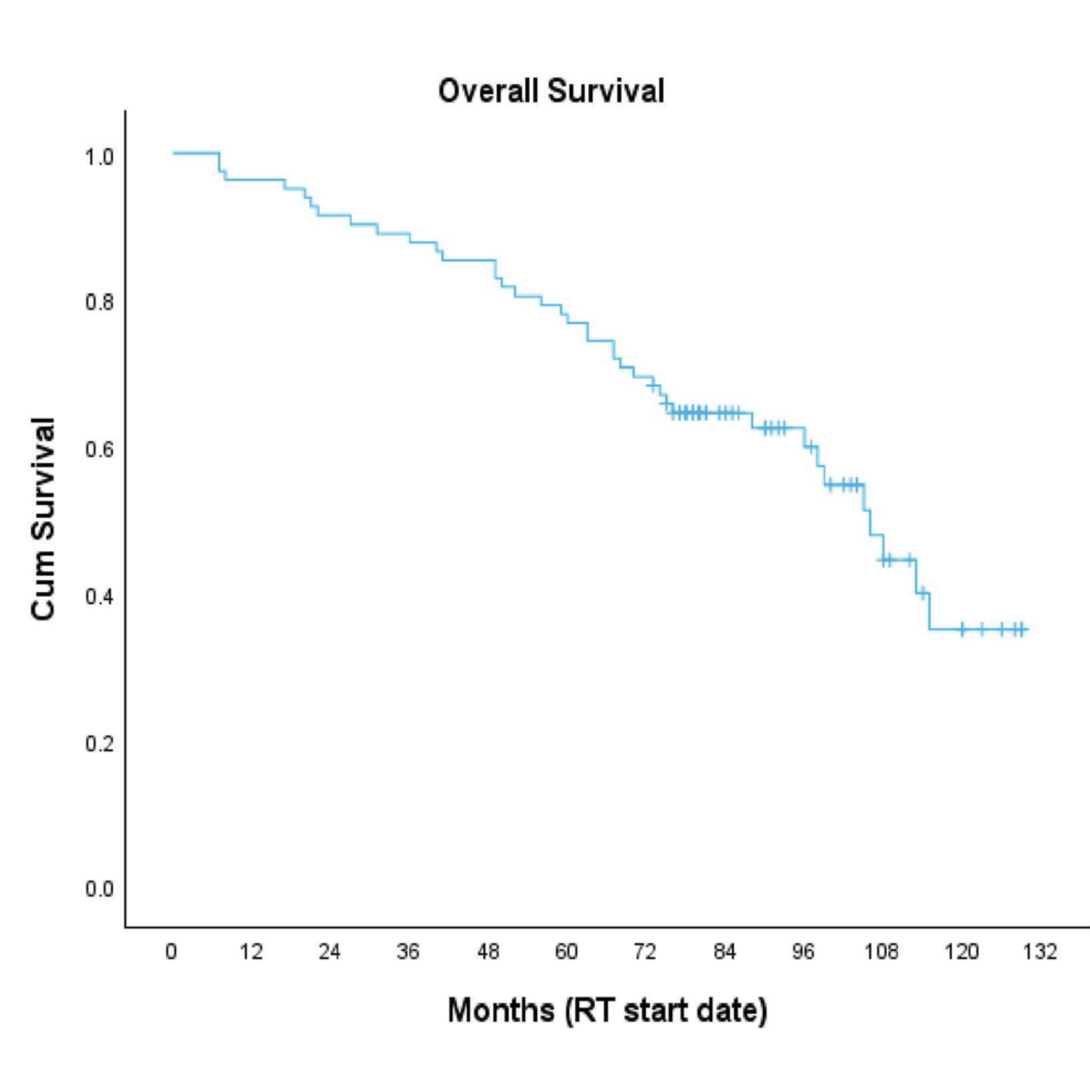
Kaplan-Meier curve for OS OS, overall survival; RT, radiotherapy [14]

Thirty-eight patients (46%) died overall. Of these, eight (10% of all 82 patients; 21% of deceased patients) died from prostate cancer, while the remaining 30 (79% of deceased patients) died of unrelated causes. The five-year OS rate was 77%, and the median OS was 106 months (Figure 4).

## Discussion

In this retrospective study of prostate cancer patients treated with radical radiotherapy at the age of 80 or older, five-year BFS, MFS, and OS were 77%, not reached, and 77%, respectively. Considering that most guidelines recommend limiting screening and treatment to men with a life expectancy of at least 10 years, our data show a median OS of approximately nine years for treated patients. This suggests good initial selection of patients deemed suitable for treatment in a cohort of older men, in whom identifying those with long life expectancy is challenging. Since all patients were at least 80 years old with nonmetastatic malignancy, their CCI scores were at least 6 [11].

The data indicate that a notable proportion of patients died of prostate cancer despite having received radical radiotherapy at the age of 80 or older. With a median follow-up of six years, 10% of the entire cohort and 21% of deceased patients had died from prostate cancer. This was a highly selected cohort of patients with low comorbidity burden and good performance status, and with localized high-risk disease, in contrast to the PSA-screening-detected cohort of men aged 50-69 years with predominantly low- to intermediate-risk disease in the ProtecT trial.

The findings also show that a significant proportion of patients did not require subsequent palliative hormone therapy (five-year HFS was 86%). This is an important endpoint, as palliative hormone therapy is continuous and affects quality of life. Although we do not have comparative data to demonstrate whether the proportion is lower in patients who did not receive radical radiotherapy, the ProtecT trial showed that therapeutic intervention reduced the incidence of metastases, local progression, and long-term hormone therapy compared with active monitoring [3]. Evidence suggests that men aged ≥70 years with localized prostate cancer have a higher incidence of high-risk disease than younger patients, which increases the likelihood of disease progression and the need for subsequent local or lifelong systemic therapy. In this context, a limited course of initial radical treatment may decrease the burden of prolonged therapy later in life, even if it does not necessarily improve OS. Long-term hormone therapy is associated with complications such as metabolic syndrome, cardiovascular disease, osteoporosis, obesity, and sarcopenia, risks that are expected to be higher than those of a short course of therapy initiated earlier in life. This is supported by evidence that adverse effects accumulate with treatment duration and that older age increases susceptibility due to baseline physiological decline [15-17].

The CHHiP trial changed practice in the UK, with most centers now using 60 Gy in 20 fractions as the standard radiotherapy dose fractionation. The trial reported five-year hormone-free and OS rates of 95% and 95%, respectively [18]. Our data showed comparable outcomes of 86% and 77%, respectively. These differences can be attributed to the fact that our cohort was older (median age, 81 years vs. 69 years) and had higher-risk disease (88% vs. 12%). Despite this, our results suggest that carefully selected patients aged 80 years or older can achieve favorable outcomes with radical prostate radiotherapy.

Prostate cancer generally follows a long natural history, and many guidelines recommend radical therapy only for patients likely to survive 10 years or more [19,20]. However, estimating prognosis over such a long period is difficult, as it requires integrating cancer characteristics, competing comorbidities, and functional status [21]. The International Society of Geriatric Oncology (SIOG) recommends tailoring treatment to individual health status rather than chronological age [22]. It suggests screening with G8 and mini-COG tools to help determine whether patients are fit, vulnerable, or frail, and thus whether they can tolerate treatment. Prostatectomy is a major undertaking for older patients, but radiotherapy is noninvasive. With modern techniques such as image-guided radiotherapy, precision targeting of the prostate reduces radiation to surrounding normal tissues, improving tolerability and enabling hypofractionation, which shortens treatment duration. Both are particularly important for older patients [23,24]. Studies have shown that comprehensive geriatric assessment (CGA) can identify patients at risk of acute radiotherapy toxicities, suggesting that routine use of CGA may further improve patient selection [19].

A limitation of this study is the absence of detailed toxicity data, as treatments were delivered as part of standard care and such data were not systematically recorded. Another limitation is the lack of a comparator cohort of patients who did not undergo radical radiotherapy, as these patients were managed by local urology teams at general hospitals and not registered at our tertiary center at the time of diagnosis, preventing follow-up. Therefore, we cannot determine whether fewer patients who underwent radical radiotherapy required palliative hormone therapy compared with those who did not, nor can we compare OS between patients managed with active monitoring (realistically, watchful waiting in this age group) versus radical radiotherapy.

Overall, older patients with prostate cancer represent a heterogeneous group, facing risks both from overtreatment of frail patients and undertreatment of fit patients [25]. Higher competing risks in frail patients should not justify withholding curative treatment from fitter patients, as uncontrolled cancer can later become more difficult to manage. Treatment decisions should be guided not by chronological age but by disease characteristics, comorbidities, and individual patient preference [26].

## Conclusions

Prostate cancer patients treated with radical radiotherapy at the age of 80 years or older can achieve favorable BFS and HFS despite high-risk disease. These outcomes are meaningful for quality of life, as a substantial proportion of patients may still die of prostate cancer despite radical treatment. Age alone should not preclude consideration of radical radiotherapy for localized disease that, if left untreated, could cause morbidity from progression and complications requiring more burdensome future treatment.
